# Hereditary Predispositions to Myelodysplastic Syndrome

**DOI:** 10.3390/ijms17060838

**Published:** 2016-05-30

**Authors:** Sarah A. Bannon, Courtney D. DiNardo

**Affiliations:** 1 Department of Clinical Cancer Genetics, The University of Texas M.D. Anderson Cancer Center, 1515 Holcombe Blvd, Houston, TX 77030, USA; sabannon@mdanderson.org; 2 Department of Leukemia, The University of Texas M.D. Anderson Cancer Center, 1515 Holcombe Blvd, Houston, TX 77030, USA

**Keywords:** MDS, germline, predisposition, hereditary, familial, genetic counseling

## Abstract

Myelodysplastic syndromes (MDS) are heterogeneous clonal hematopoietic disorders characterized by ineffective hematopoiesis, bone marrow dysplasia, and peripheral cytopenias. Familial forms of MDS have traditionally been considered rare, especially in adults; however, the increasing availability of somatic and germline genetic analyses has identified multiple susceptibility loci. Bone marrow failure syndromes have been well-described in the pediatric setting, e.g., Fanconi anemia (FA), dyskeratosis congenita (DC), Diamond–Blackfan anemia (DBA), and Shwachman–Diamond syndrome (SBS), hallmarked by clinically-recognizable phenotypes (e.g., radial ray anomalies in FA) and significantly increased risks for MDS and/or acute myeloid leukemia (AML) in the setting of bone marrow failure. However, additional families with multiple cases of MDS or AML have long been reported in the medical literature with little known regarding potential hereditary etiologies. Over the last decade, genomic investigation of such families has revealed multiple genes conferring inherited risks for MDS and/or AML as the primary malignancy, including *RUNX1*, *ANKRD26*, *DDX41*, *ETV6*, *GATA2*, and *SRP72*. As these syndromes are increasingly appreciated in even apparently *de novo* presentations of MDS, it is important for hematologists/oncologists to become familiar with these newly-described syndromes. Herein, we provide a review of familial MDS syndromes and practical aspects of management in patients with predisposition syndromes.

Myelodysplastic syndromes (MDS) are clonal neoplastic blood disorders characterized by ineffective hematopoiesis, peripheral cytopenias, bone marrow dysplasia, and an increased risk of acute myeloid leukemia (AML). Surveillance, Epidemiology and End Results (SEER) data support the notion that MDS is an increasingly prevalent disease of predominantly advanced age (median age at onset is 71 years), while MDS in childhood, adolescence and young adulthood is rare. MDS has historically been categorized as *de novo* or primary MDS, or secondary or therapy-related MDS arising from previous treatment with cytotoxic therapy (ionizing radiation, alkylating agents, and DNA topoisomerase II inhibitors) [1].

Within pediatric oncology, there is an understanding of rare inherited predispositions to primary MDS associated with bone marrow failure syndromes such as Fanconi anemia (FA), dyskeratosis congenita (DC), and Schwachman–Diamond syndrome (SBS) in children. However, approximately 10%–20% of individuals with FA and nearly 50% of individuals with DC are diagnosed as adolescents or young adults. Within apparently sporadic primary MDS in young adults, or those with familial clustering of MDS, an underlying germline susceptibility to MDS is likely more common than previously considered. As genetic sequencing has become increasingly integrated into clinical practice, clearly defined syndromes have emerged, termed familial MDS/acute myeloid leukemia (AML) predisposition syndromes [2]. In addition, somatic molecular testing for MDS prognostication is not yet routine in the evaluation of newly diagnosed MDS patients but is a rapidly growing mechanism for clinical evaluation that can also indicate a potential underlying predisposition syndrome. Recognizing patients with potential hereditary syndromes and referring them for genetic evaluation and genetic counseling not only can provide valuable insights for treatment of their disease but also education, risk assessment, and psychosocial support for these individuals and their family members. The increasing awareness of these conditions coupled with efforts to refer for genetic counseling and genetic testing has revealed that as high as 10% of individuals with hematologic malignancies may carry a germline susceptibility—much higher than previously thought.

## 1. Familial Myelodysplastic Syndromes (MDS)/Acute leukemia (AL) Predisposition Syndromes

To date, there are seven well-defined single-gene loci that, when mutated, predispose to an increased lifetime risk of primary MDS and/or AML (Table 1). Individuals who carry mutations within these genes often have other concomitant characteristics that can be subtle or even absent, particularly in those patients diagnosed in adulthood. Additionally, multiple families with a clear clustering of MDS and/or AML do not harbor germline mutations in these described genes, indicating that additional pathogenic loci remain to be identified. Table 1 summarizes the seven known single-gene predispositions to inherited MDS, as well as the two most common “pediatric” bone marrow failure syndromes which lead to an increased risk of adult-onset MDS. With the exception of *CEBPA* germline mutations, which appear to confer an increased risk of only AML, these syndromes overlap substantially in their associated risks of MDS, AML and thrombocytopenia, making them difficult to distinguish based on clinical characteristics alone. Owing to the risk of isolated AML without the development of MDS, *CEBPA* germline mutations are beyond the scope of this review.

## 2. Familial Platelet Disorder with Propensity to Myeloid Malignancy (FPD/AML)

Familial platelet disorder with propensity to myeloid malignancy is an autosomal dominant familial MDS/AML syndrome caused by inherited mutations in the hematopoietic transcription factor *RUNX1*. *RUNX1* is located at 21q22 and causative mutations are most often frameshift, nonsense, or deletion mutations that result in premature protein truncation; missense mutations in the DNA binding domain are also reported. Most characterized germline mutations in *RUNX1* lead to protein loss-of-function or confer dominant-negative effects to the remaining *RUNX1* allele [12]. Clinically, patients with FPD/AML often present with life-long thrombocytopenia and aspirin-like functional platelet defects [3]. The degree of thrombocytopenia in individuals with *RUNX1* mutations is typically mild to moderate and can vary widely even within affected families from individuals with a normal platelet count, to severe thrombocytopenia, to childhood MDS/AML at the time of first evaluation. The lifetime risk of MDS or acute leukemia is estimated to be 35%–40%, with an average age at diagnosis of 33 years (range: 6–76 years) in described patients [13]. The hematologic malignancies described in FPD/AML patients include MDS, AML, and T-cell acute lymphoblastic leukemia (ALL). Currently, affected individuals do not typically require treatment for thrombocytopenia in the absence of clinical bleeding, although care must be taken in the setting of surgical procedures including obstetrical care where bleeding can be out of proportion to the platelet count due to platelet dysfunction. No clinical or laboratory markers are currently available to predict when a patient with FPD/AML will develop an overt malignancy; however, recent data suggests clonal hematopoiesis can be detected in >80% of asymptomatic FPD/AML individuals by age 50, and may provide a future means of disease surveillance [14]. For individuals without hematologic malignancy who harbor germline *RUNX1* mutations, current recommendations include a bone marrow biopsy at baseline with cytogenetic analysis, followed by complete blood count (CBC) and clinical exams at regular intervals. Any time significant changes in the CBC are identified, a repeat marrow should be performed. In the event that a *RUNX1* mutation carrier develops acute leukemia or MDS requiring allogeneic stem cell transplantation, genetic testing should be performed urgently on human leukocyte antigen (HLA)-matched related donors in whom the *RUNX1* status is not previously known, and those who also carry the mutation should not be used as donors. Adverse outcomes including donor-derived leukemia and failure to engraft have been reported [2].

## 3. Thrombocytopenia 2

Associated with germline mutations in the 5′ untranslated region of the gene Ankyrin Repeat Domain 26 (*ANKRD26*) on chromosome 10p12, Thrombocytopenia 2 is an autosomal dominant disorder that is characterized by moderate thrombocytopenia with or without bleeding propensity, similar to FPD/AML. In the initial description of *ANKRD26* mutations, Pippucci *et al.* [4] sequenced the coding region of a previously identified candidate locus at 10p (THC2) for inherited thrombocytopenia, revealing only polymorphisms. Further analysis of the 3′ and 5′ untranslated regions (UTRs) revealed eight heterozygous single nucleotide substitutions within the 5′ UTR in eight different families, clearly segregating with the linked haplotype within the pedigrees. Interestingly, a 5′ UTR *ANKRD26* mutation was later found in the Italian kindred initially reported with an *ACBD5* mutation at the THC2 locus, revealing the *ACB5* to be a private rare variant linked to the THC2 locus, rather than being the cause of inherited thrombocytopenia [4]. In a recent study of 78 affected individuals from 21 families with familial Thrombocytopenia 2, the average platelet count was 48,000/mL, with a normal platelet volume and decreased alpha-granule content leading to a pale platelet appearance, decreased platelet surface membrane glycoprotein Ia (GPIa), elevated thrombopoietin levels, and variable platelet aggregation defects [15]. In affected individuals, bone marrow morphology can demonstrate dyskmegakaryopoeisis with hypolobulated micromegakaryocytes at baseline, presenting a diagnostic challenge for hematopathologists to appropriately distinguish individuals with germline *ANKRD26* mutations *versus* dysmegakaryopoiesis related to development of MDS [15]. The prevalence of Thrombocytopenia 2 is not well described; an inherited thrombocytopenia registry identified *ANKRD26* mutations in 23 cases out of 215 individuals (11%) [16]. Individuals with *ANKRD26* are clinically difficult to distinguish from those with FPD/AML and surveillance recommendations are similar, including a bone marrow biopsy with cytogenetics at diagnosis and physical exam and blood work at regular intervals. Similar to other inherited predispositions to leukemia, care should be taken to perform genetic testing in family members should an allogeneic stem cell transplant be required [2].

## 4. Familial AML with Mutated *DDX41*

One of the most recently described inherited susceptibility loci for myeloid neoplasms, *DDX41* germline mutations, located on chromosome 5q, are associated with autosomal dominant familial MDS/AML. Within the nearly 17 pedigrees that have been described to date, a recurrent mutation, p.D140Gfs*2, appears to account for the majority of germline mutations. *DDX41* mutations result in an increased lifetime risk of myeloid neoplasms including MDS, AML, and chronic myeloid leukemia (CML), although notably after a long latency, with an average age of disease onset of 61 years [17]. This average age at diagnosis falls within the expected age range of MDS/AML in the general population, thus it may be clinically difficult to distinguish patients with *de novo* MDS/AML from those with a germline predisposition due to a germline mutation in *DDX41*, and this syndrome may be particularly under-diagnosed. Individuals with germline *DDX41* mutations typically develop normal karyotype, high grade myeloid neoplasms, including a variety of MDS subtypes (refractory cytopenias with multilineage dysplasia, refractory anemia with excess blasts, chronic myelomonocytic leukemia (CMML), and 5q-syndrome), all with apparently poor prognosis. Myeloid malignancies in patients with *DDX41* mutations often demonstrate an acquired somatic mutation in the wild-type *DDX41* allele, suggesting that it may act as a tumor suppressor [18]. The prevalence of *DDX41* germline mutations is not known; however in a study screening 1000 myeloid neoplasm cases, *DDX41* were identified in 1.5%, half of which were germline [5], suggesting that identification of a *DDX41* mutation should prompt consideration of germline analysis.

Unlike the majority of described syndromes predisposing individuals to MDS, *DDX41*-related malignancies have no apparently preceding clinical signs or symptoms as harbingers of the increased risk for hematologic malignancy, outside of a significant family history. Even then, with the average age of diagnosis in the seventh decade of life, other affected family members may have yet to develop a hematologic malignancy to suggest this inherited syndrome. The difficulty of identifying *DDX41* mutation carriers prior to diagnosis in the general population makes surveillance of unaffected individuals a rare occurrence to date. In families with known *DDX41* mutations, like other predisposition syndromes, a bone marrow biopsy is recommended at diagnosis with cytogenetic analysis and CBC at regular intervals [2].

## 5. Thrombocytopenia 5

Thrombocytopenia 5 is an inherited autosomal dominant MDS/AML predisposition syndrome associated with moderate thrombocytopenia, with or without a clinical bleeding propensity. Similar to *RUNX1* and *ANKRD26* mutation carriers, individuals with mutations in Ets variant 6 (*ETV6*)—associated with Thrombocytopenia 5—present with a variable degree of thrombocytopenia and mild-to-moderate bleeding tendencies. Thrombocytopenia 5 is caused primarily by missense mutations in the gene *ETV6*, located on chromosome 12p, which appear to have a dominant negative function, disrupting the nuclear localization of the *ETV6* protein and resulting in reduced expression of platelet-associated genes. Individuals with germline *ETV6* mutations are reported to be at increased risk for all hematologic malignancies, including MDS, AML, CMML, B-lymphoblastic leukemia, and plasma cell myeloma. Early-onset colorectal cancer has also been reported in a small number of individuals to date [6]. As with other hereditary familial platelet disorders, individuals carrying germline *ETV6* mutations are recommended to undergo bone marrow biopsy with cytogenetic analysis at diagnosis with CBC screening at regular intervals; and care should be taken to avoid using family members who also carry the mutation for stem cell transplantation due to adverse outcomes [2].

## 6. Familial MDS/AML with Mutated *GATA2* (*GATA2* Deficiency)

*GATA2* deficiency, also known as familial MDS/AML with mutated *GATA2*, is a clinically heterogenous predisposition to MDS. Individuals with germline *GATA2* mutations on chromosome 3q21 can present without any hematopoietic or organ system involvement prior to the development of MDS or AML; however, there are two distinct syndromic presentations that can be seen with this particular syndrome. Emberger syndrome describes *GATA2* deficiency clinically characterized by primary lymphedema, sensorineural hearing loss, cutaneous/extragenital warts, and a low CD4/CD8 T-cell ration with a predisposition to MDS/AML. The MonoMac syndrome, also related to germline *GATA2* deficiency, is characterized by dendritic cells, monocytes, and B/NK cell deficiencies, leading to the development of atypical mycobacterial or fungal infections, pulmonary alveolar proteinosis, and MDS/AML predisposition. The phenotypes can overlap and, because they share the same underlying genetic etiology, are considered part of the same autosomal dominant genetic disorder with variability [7]. Individuals with *GATA2* germline mutations are at significantly increased lifetime risk of MDS/AML; approximately 70% by a median age of onset of 29 years (range 0.4–78). Presenting symptoms among 57 patients with germline *GATA2* deficiency from the National Institutes of Health (NIH) cohort were described, including viral infections in 23%, disseminated non-tuberculosis mycobacterial infections in 28%, MDS/AML in 21%, lymphedema in 9%, and invasive fungal infections in 4% [19].

Management and surveillance of individuals with *GATA2* deficiency often involve a multidisciplinary care team given the multi-organ involvement. The high incidence of MDS/AML in these individuals also warrants close evaluation with regular peripheral blood testing for both signs of worsening immunodeficiency as well as monitoring blood counts. A bone marrow biopsy, like with all of these heritable syndromes, is recommended at baseline with cytogenetic analysis and repeated with any changes in CBC worrisome for developing MDS/AML.

## 7. Familial Aplastic Anemia/MDS with *SRP72* Mutation

Germline mutations in the ribonucleoprotein complex gene *SRP72* (Signal Recognition Particle 72 kDa) have been identified as a rare cause of familial MDS and bone marrow failure. Two pedigrees with autosomal dominant MDS and aplastic anemia have been reported. In both families, MDS developed in adulthood. Given the rarity of these germline mutations, little is known regarding the incidence, lifetime risk for aplastic anemia (AA)/MDS and/or targeted clinical management guidelines of these families [8].

## 8. Bone Marrow Failure Syndromes

Bone marrow failure syndromes are typically associated with onset of MDS, AA or AML in childhood or young adulthood. While the majority of individuals with bone marrow failure syndromes will have syndromic phenotypic abnormalities such as multiple congenital anomalies or pancreatic dysfunction at presentation, there can be subtle (or completely lacking) phenotypic presentations resulting in a delayed diagnosis into adulthood, at the time of development of malignancy. Individuals with Fanconi anemia and dyskeratosis congenita are at significantly increased risk for treatment-related toxicities when treated with cytotoxic therapies, particularly in the context of stem cell transplantation, and also at risk for treatment-induced malignancies [20].

## 9. Fanconi Anemia

Estimated at 1:360,000 births, Fanconi anemia (FA) is a rare, autosomal recessive or X-linked inherited predisposition to bone marrow failure. Accompanied primarily by congenital limb anomalies including absent thumbs and other radial ray defects, Fanconi anemia is characterized by increased chromosomal fragility and breakage when treated with cross-linking agents, specifically diepoxybutane (DEB) or mitomycin C (MMC). Progressive bone marrow with pancytopenia typically appears in the first decade, and by age 50, the cumulative incidence of bone marrow failure is estimated to be 90%. The incidence of hematologic malignancies (primarily MDS/AML) is 10%–30% and there is also an increased risk of solid tumors, particularly squamous cell carcinomas of the head and neck. A sizeable subset of individuals with FA, 40%, lack physical abnormalities associated with the disease and are also less likely to develop early-onset bone marrow failure. In this subset of patients, there is a higher likelihood to develop MDS/AML and other early-onset solid tumors as a presenting symptom of the underlying inherited syndrome [21]. Additionally in this subset of young patients with apparently *de novo* MDS, a characteristic pattern of somatic chromosomal translocations can indicate underlying Fanconi anemia, of particular importance as these individuals require particular care such as reduced-intensity conditioning for stem cell transplantation [22].

Fanconi anemia is caused by homozygous or compound heterozygous mutations in the 19 FA complementation groups, FANCA-FANCP [23]. Mutations in FANCA are the most common, accounting for 60%–70% of cases of FA, followed by FANCC, accounting for 14%. The remaining 13 complementation groups account for 1%–3% of cases each. Notably, *BRCA1*, *BRCA2*, *PALB2*, and *RAD51C* are each part of the FA complementation groups and, when inherited as heterozygous mutations, are associated with autosomal dominant predispositions to solid tumor development, particularly hereditary breast and ovarian cancer syndrome [24,25]. Mutations in *PALB2* and *RAD51C* are associated with moderate risks for breast cancer and ovarian cancer, respectively. Thus, parents and siblings of an individual with FA caused by one of these specific complementation groups who are heterozygous carriers should also be referred for genetic counseling and management of solid tumor risks, even though they do not have a diagnosis of Fanconi anemia [26].

## 10. Dyskeratosis Congenita/Telomeropathies

Dyskeratosis congenita (DC) is a telomere biology disorder originally characterized by a diagnostic triad of dysplastic nails, lacy reticular skin pigmentation, and oral leukoplakia; however, these features are not present in all individuals with DC and may or may not develop over time [11]. Individuals with DC are at increased risk for bone marrow failure (BMF), MDS, or AML, solid tumors (typically squamous cell carcinomas of the head, neck, anogenital tract), and pulmonary fibrosis. The median age at onset of first malignancy is 37 years (range 25–44), with a specific study reporting the median age of onset of MDS as 35 years (range 19–61) [27]. DC is characterized by very short telomeres, defined as less than 1% of age-matched normal controls, determined by multicolor flow cytometry fluorescence *in situ* hybridization (flow-FISH) on white blood cell subsets. Eight genes have been identified to cause DC: *CTC1*, *DKC1*, *TERC*, *TERT*, *TINF2*, *NHP2*, *NOP10*, and *WRAP53*, although pathogenic germline mutations can be detected in only ~50% of individuals with a clinical diagnosis of DC. *TERT* and *TERC* germline mutations have been associated with acquired aplastic anemia, idiopathic pulmonary fibrosis, and adult-onset disease [28]. DC should be specifically considered as a potential etiology of BMF in individuals in whom FA has been excluded, and/or with the presence of head/neck/anogenital squamous cell carcinoma in individuals younger than 50 without risk factors. Telomere length analysis in total leukocytes and a panel of six leukocyte subsets (granulocytes, naïve T-cells, memory T-cells, B-cells, and NK cells) by flow-FISH is recommended in these patients to detect an underlying diagnosis of DC [29].

Hematopoietic stem cell transplant (HSCT) is the only curative option for severe BMF or MDS/leukemia in patients with DC. However, care must be taken given an increased rate of HSCT-related complications in patients with DC, including increased risk of graft failure, graft *vs.* host disease, sepsis, pulmonary fibrosis, hepatic cirrhosis, and veno-occulsive disease caused in part by the underlying pulmonary and liver disease. Therefore, long-term survival of individuals with DC following standard stem cell transplant (SCT) regimens has been poor [30].

## 11. Identifying Patients with Potential Hereditary Predispositions and Genetic Counseling

**Figure 1:**
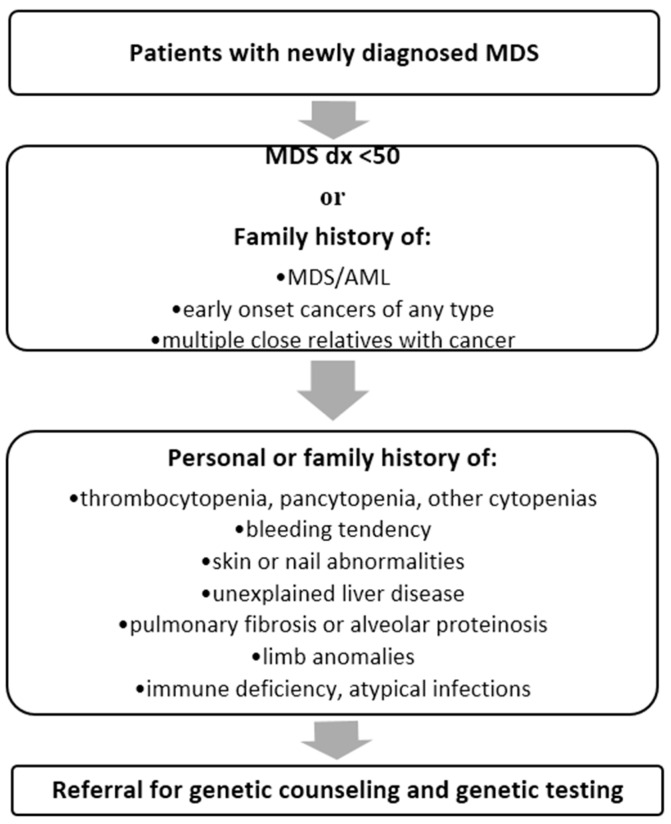
Referral algorithm for genetic evaluation of hereditary MDS (myelodysplastic syndromes).

Recognizing individuals with MDS who may harbor germline mutations can be significantly enhanced by obtaining a careful medical and family history. Guidelines for the clinical detection of familial MDS syndromes have been proposed, and Churpek *et al.* propose an algorithm for screening newly diagnosed myeloid malignancy patients for further referral for genetic counseling and genetic testing (Figure 1) [2].

In addition to the clinical criteria proposed in Figure 1, in patients with MDS for whom routine molecular testing is performed for diagnostic and/or prognostic purposes and identifies a mutation in any of the known causative genes of familial MDS syndromes, consideration of germline evaluation is essential. The clinical phenotypes of these syndromes are rapidly expanding and these criteria may not encompass all individuals with a potential hereditary predisposition syndrome. Particularly related to germline *DDX41* mutations, age at diagnosis may be over the age of 50, indicating that family history is an essential tool for identifying potential hereditary disease. In individuals with MDS meeting any of the listed criteria, or in whom somatic testing raises concern for a potential underlying syndrome, referral to genetic counseling is highly recommended.

Comprehensive genetic evaluation and counseling involves a thorough review of an individual’s personal medical and family histories, including review of somatic molecular testing and review of medical diagnoses in family members. Through the process of genetic counseling, individuals with MDS are educated regarding the known hereditary etiologies for hematologic malignancies, provided a personalized risk assessment of the likelihood of a hereditary predisposition within his/her family, and in most cases, offered genetic testing to investigate or confirm the possibility of a germline mutation. As part of genetic counseling, psychosocial assessment and counseling is also provided surrounding psychological concerns unique to hereditary cancer predisposition syndromes, including coping with a diagnosis of cancer, uncertainty, and fear and guilt of having potentially inherited and/or passed a cancer predisposition to family members or children. Psychological concerns unique to hereditary hematologic malignancies also include careful counseling regarding the (current) inability to prevent the future development of MDS or leukemia, as well as facilitating coping with a potential predisposition to a disease that has a rapid onset, occasionally without any preceding signs/symptoms, and with a high mortality rate. Because these mutations are, by definition, inherited, genetic counseling is also of paramount importance for the facilitation of genetic testing in family members who are also at-risk to inherit these hereditary syndromes. Genetic counseling helps to identify which family members are at-risk, their likelihood of inheriting the mutation, pre-test counseling for family members about the risks, benefits, and limitations of genetic testing, facilitating the test in family members, and providing post-test counseling about the results, their implications, and referrals for increased surveillance and management as well as psychosocial support.

## 12. Genetic Testing

Specimen selection for genetic testing in individuals with MDS is of utmost importance. While peripheral blood is the typical source of DNA for genetic testing in solid tumor patients, it is typically contaminated with tumors in individuals with MDS and other hematologic malignancies. Therefore, genetic testing on blood is somatic testing and cannot accurately assess the germline, particularly given the fact that many genes which confer a germline predisposition may also be somatically mutated in MDS. Skin fibroblasts are the recommended source of germline DNA for individuals with hematologic malignancies for germline analysis. Standard skin punch biopsies can be performed easily in the clinic with minimal risk, or at the time of bone marrow biopsy. Fibroblasts are cultured in a Clinical Laboratory Improvement Amendments (CLIA)-certified environment prior to genetic testing, which can take up to 2–4 additional weeks.

Germline genetic testing is clinically available for the majority of the genes discussed through CLIA-certified clinical molecular genetic testing laboratories, from large diagnostic companies to academic hospital-based laboratories. In most cases, insurance companies cover the cost of genetic testing, which typically ranges from $1000–$3000 USD. Given the phenotypic overlap of the known syndromes (at least four are associated with thrombocytopenia), a panel-based approach to genetic testing is typically preferred, which offers the ability to analyze multiple genes simultaneously. Laboratories that offer genetic testing for these genes are listed through GeneTests [31], an online searchable database for genetic testing labs. Next-generation sequencing is used in the majority of cases, utilizing high-throughput sequencing to generate the sequence, often complemented by array-comparative genomic hybridization (CGH) to evaluate for deletions and duplications. Due to the requirement of DNA from cultured skin fibroblast culture in the majority of hematologic malignancy cases, the turnaround time for genetic testing in this population ranges from six to eight weeks. Clinical genetic test reports characterize germline alterations according to the American College of Medical Genetics standards and guidelines for the interpretation of sequence variants [32]. Germline alterations are reported with standard terms and nomenclature and their pathogenicity is categorized according to specific available lines of evidence. Clinically actionable results are reported as either “pathogenic” or “likely pathogenic”. Due to the relative novelty of the majority of the genes implicated in hereditary predispositions to MDS and AML, less is known about unique variants seen for the first time. Germline alterations with insufficient or conflicting evidence regarding pathogenicity are reported as “variants of uncertain significance” (VUS). The presence of VUS in a gene associated with an inherited predisposition to MDS/AML does not necessarily confer a hereditary risk. Genetic testing in other family members when an uncertain result is present should be performed with great care, and is highly recommended to be performed in the context of genetic counseling.

## 13. Conclusions and Implications for Practice

Multiple hereditary predispositions to MDS have been recently discovered, particularly since the advent of easily-accessible panel-based molecular genetic testing is now integrated into the evaluation, prognostication, and treatment of patients with MDS. Individuals with inherited predispositions to MDS/AML are likely not as rare as previously thought and have unique treatment considerations, particularly in regards to allogeneic SCT. The treatment considerations and need for familial screening are of such paramount importance in clinical practice that the forthcoming World Health Organization (WHO) classification guidelines will include these hereditary malignancies as novel entities [33]. Clinicians should be aware of the signs and symptoms of hereditary predispositions to hematologic malignancies, and obtain a careful family and medical history in all patients with MDS and AML to identify patients who may be appropriate for further genetic counseling and testing. In particular, patients diagnosed with MDS or bone marrow failure syndrome at a young age, a strong personal and/or family history of malignancy, and/or the identification of mutations (*i.e.*, *GATA2*, *CEBPA*, *DDX41*, *RUNX1*) on next-generation sequencing (NGS)-based somatic cancer panels that may be germline events, should prompt a referral to genetic counseling for evaluation of a possible hematologic malignancy predisposition syndrome.

## Figures and Tables

**Table 1:** Familial myelodysplastic syndromes (MDS)/acute leukemia (AL) predisposition syndromes.

Syndrome	Gene	Inheritance	Heme Malignancy	Other Associated Abnormalities	Reference
Familial platelet disorder with propensity to myeloid malignancies	*RUNX1*	AD	MDS/AML/T-cell ALL	Thrombocytopenia, bleeding propensity, aspirin-like platelet dysfunction	[3]
Thrombocytopenia 2	*ANKRD26*	AD	MDS/AML	Thrombocytopenia, bleeding propensity	[4]
Familial AML with mutated DDX41	*DDX41*	AD	MDS/AML, CMML	None	[5]
Thrombocytopenia 5	*ETV6*	AD	MDS/AML, CMML, B-cell ALL, multiple myeloma	Aplastic anemia	[6]
Familial MDS/AML with mutated GATA2	*GATA2*	AD	MDS/AML/CMML	Neutropenia, monocytopenia, MonoMAC syndrome, Emberger syndrome	[7]
Familial aplastic anemia with SRP72 mutation	*SRP72*	AD	MDS	Aplastic anemia	[8]
Familial AML with mutated CEBPA	*CEBPA*	AD	AML	None	[9]
Fanconi anemia	Complementation Groups	AR, X-linked	MDS, AML	Pancytopenia, macrocytic anemia, congenital malformations	[10]
Telomeropathies (dyskeratosis congenita)	*TERC*, *TERT*, others	AD, AR	MDS/AML	Macrocytosis, aplastic anemia, oral leukoplakia, dysplastic nails, lacy skin rash	[11]

AD, Autosomal dominant; MDS, myelodysplastic syndrome; AML, acute myeloid leukemia; ALL, acute lymphoblastic leukemia; CMML, chronic myelomonocytic leukemia; AR, autosomal recessive.
